# Changing Sleep Architecture through Motor Learning: Influences of a Trampoline Session on REM Sleep Parameters

**DOI:** 10.3390/life14020203

**Published:** 2024-01-31

**Authors:** Daniel Erlacher, Daniel Schmid, Stephan Zahno, Michael Schredl

**Affiliations:** 1 Institute of Sport Science, University of Bern, CH-3012 Bern, Switzerland; daniel.schmid@students.unibe.ch (D.S.); stephan.zahno@unibe.ch (S.Z.); 2 Central Institute of Mental Health, Medical Faculty Mannheim, Heidelberg University, 68159 Mannheim, Germany; michael.schredl@zi-mannheim.de

**Keywords:** motor memory consolidation, sleep architecture, REM sleep, procedural memory, trampoline, gross motor learning

## Abstract

Previous research has shown that learning procedural tasks enhances REM sleep the following night. Here, we investigate whether complex motor learning affects sleep architecture. An experiment in which twenty-two subjects either learned a motor task (trampolining) or engaged in a control task (ergometer) was carried out in a balanced within-group design. After an initial laboratory adaptation night, two experimental nights were consecutive. The results indicate that learning a motor task had an effect on REM sleep parameters and, therefore, support the hypothesis that learning a procedural skill is related to an increase in REM sleep parameters. However, the statistical effect on REM sleep is smaller than found in previous studies. One might speculate that the motor learning was not intense enough compared to other studies. For sports practice, the results suggest that REM sleep, which is particularly rich in the morning, plays an important role in motor memory consolidation. Thus, this phase should not be interrupted after complex motor skill learning sessions. In future studies, other motor tasks should be applied.

## 1. Introduction

Sleep does seem to play a role in the process of learning and motor memory [1]. However, the multifaceted nature of sleep and motor memories led to an equivocal state-of-the-art in the last two decades [2]. Meta-analytical evidence regarding motor memory consolidation and sleep indicates that sleep has a small to medium beneficial effect on task performance after sleep compared to a corresponding wake interval [3]. Furthermore, certain sleep stages have been linked to motor performance after sleep in several correlative studies, especially with classic experimental tasks like finger-tapping (for an overview, see [2]). However, the correlative nature of this research precludes the formulation of causal effects. Moreover, the generalization of findings based on isolated lab tasks might be limited when it comes to the highly complex skills that we see in sports practice [4]. A promising alternative is to observe the influence of complex skill learning on the following night’s sleep and its architecture compared with a baseline night. This approach could strengthen our knowledge of the electrophysiological basis of motor memory consolidation and help us to understand what in sleep leads to motor memory consolidation.

Until now, only a few studies have investigated how learning a sports motor skill influences the architecture of the following night’s sleep [5,6,7,8]. Using a multitask research strategy (pursuit rotor, simple tracing task, ball-and-cup game, and the operation game), Fogel and Smith [6] found learning-dependent changes in stage 2 sleep duration and sleep spindles during stage 2 sleep for the experimental but not for the control group while no other changes in sleep architecture were observed [6]. However, since a multiple task strategy was used, it is impossible to dissect the influence of the different tasks used in this study. Additionally, the control group did not participate in any physical activity and watched videos. In a balanced within-subject study, participants had to learn performing a snakeboarding task (an adaptation of a skateboard in which one is propelled forward by performing snake like movements) [5]. Additionally, it was controlled for the first-night effect [9] by introducing an adaptation night. No differences between the experimental task and a control task—a bicycle ergometer session—could be found for REM sleep parameters [5]. Using a 2 h daytime nap and a three-ball cascade juggling task alternation of slow oscillations, delta wave, and sigma wave spectral power were increased in NREM sleep after a post-learning nap in comparison with a baseline nap [8]. Also using a 2 h daytime nap increased sleep spindle activity and longer REM duration were observed after a learning session on an inverse steering bicycle. However, it should be noted that these changes in sleep architecture were negatively correlated with task improvement [7].

Another study conducted by Blischke et al. [10] investigated the influence of sleep on a submaximal countermovement jump, where participants were required to generate a submaximal vertical force impulse equivalent to precisely 60% of their individual maximum. The study examined two groups, both of which underwent training sessions 12 h apart and returned for performance testing 12 h and 24 h after their respective training sessions. Interestingly, no discernible difference in performance on this gross motor task was observed between individuals who had experienced sleep and those who had remained awake. However, on a re-analysis of the same study condition applying a sleep-related gain calculation, a significant difference was found. In a more recent study, participants were randomly assigned to either a sleep group or a wakefulness group. They were then trained on an arm-coordinated reaching task. Notably, gross motor skill performance showed improvement in both groups following a night of sleep, but there was no such improvement after a day of wakefulness [11]. 

So far, most studies investigating the influence of skill learning on sleep architecture used relatively gross motor tasks. These experimental tasks often require only the learning of one single movement. However, in sport practise, one is often required to learn various movements in the same sport discipline like, for example, different jumps on a trampoline. Such a learning protocol was implemented in a study by Buchegger et al. [12] in which participants of the experimental condition participated in a 13-week basic trampolining course. All participants in the experimental condition were initially novices on the trampoline and participated a 2 h per week training session. After every training session, two of the participants spent one night in the sleep laboratory, during which polysomnographic recordings were performed from 12 AM to 8 AM. The procedure of the control group was the same; however, they participated in a sport course, where they trained an already known sport like football or dancing in which motor learning was not dominant. A marked change in sleep architecture was found with an increase in REM sleep from baseline, which took place one week before the sleep laboratory night (22.8% and 22.6%) was compared to the experimental nights (30.2%, 28.7%, and 27.4%). Furthermore, the difference between the experimental and control group was significant (REM sleep parameters control group: 21.2%, 24.2%, and 20.9%). However, the study has three disadvantages which should be addressed. Firstly, due to the chosen design, electrophysiological data were collected at different time points of the 13-week training programme, which could markedly influence the effect of the learning session on sleep architecture, as the learning progress was not the same for the participants. Secondly, the first night in the sleep laboratory was the baseline night. However, the first night in the sleep laboratory is often marked by decreased total sleep time and sleep efficiency and an increase in wakefulness and REM sleep latency, and thus the baseline sleep parameter might represent low baseline values. Thirdly, it is uncertain whether the control task really was free of a motor learning aspect. 

Thus, the current study aims to replicate the findings from Buchegger et al. [12] while addressing the limitations of their study and adapting the experimental setup. This was carried out by firstly optimizing the learning protocol for one session; thus, when the participants were assessed in the sleep lab, they were nearly at the same point in the learning curve. Secondly, by taking the first-night effect into account by introducing an adaptation night. Thirdly, by using a control task with increased physical activity but motor learning aspects held to a minimum. We hypothesised that in a night following learning a complex motor task more REM sleep will be observed in comparison to a night following performing a motor task without motor learning.

## 2. Materials and Methods

### 2.1. Participants

Participants were recruited at the psychological institute of the University of Heidelberg and Mannheim. Before the study participants were screened in relevant variables such as their general fitness level, experience in trampolining, ballet, and gymnastics as well as their sleep behaviours. The final sample consisted of 22 participants (N = 22, 12 female) with a mean age of 22.5 (SD = 3.9) years. None of the participants had previous experience on the trampoline, other than once at school with a time difference of at least 5 years. Written informed consent was obtained from the participants and the study was carried out in accordance with the 1964 Declaration of Helsinki. Participants received 150.00 EUR for their participation.

### 2.2. Screening

The sleep laboratory study was advertised by a notice at the University of Heidelberg and Mannheim. Interested persons were invited to contact the institute by telephone. In this telephone conversation, a screening was carried out on the basis of standardized questions regarding sleep habits and previous sports experience. The questions (from standardized questionnaires LISST and SF-B, e.g., [7,13]) included various information on sleep latency and sleep quality, as well as specific previous experience of the sports trampoline, ballet, gymnastics, etc., and general fitness. If the information provided showed no abnormalities, then the study participants were informed of the details of the study and an appointment was made.

### 2.3. Procedure

#### 2.3.1. General Procedure

Participants visited the laboratory on three occasions. The first night served as an adaptation night. Furthermore, during the adaptation night, besides the standard polysomnographic record (EEG, EOG, and EMG), nasal and oral airflow, chest and waist movements, blood saturation, and anterior tibialis electromyography were measured to diagnose potential sleep problems. The second and third night served as experimental nights with a balanced within-group design, in which participants either had to participate in a trampolining (experimental task) or a bicycle ergometer (control task) session. The sessions took place between 5 p.m. and 7 p.m. Lights off was at 11 p.m. and standard polysomnography was recorded from 23 p.m. to 7 a.m. After every night in the laboratory, participants filled in a standard sleep questionnaire regarding their subjective sleep variables [14,15]. 

#### 2.3.2. Experimental and Control Task

Trampoline: Participants had to perform various trampoline exercises with increasing difficulty levels across the exercises (basic jumps, seat drop, etc.). The time on the trampoline was 1 h overall. To reduce the physical strain six breaks of 10 min in between were implemented, leading to an overall duration of the experimental session of 2 h. After learning the basic jump types, a freestyle had to be jumped. In order to guarantee standardized conditions for all test participants and to be able to respond to the different learning rates of the test subjects, the introduction to trampolining was always conducted in the same way. In the learning protocol it was recorded after how many minutes an element was jumped at least ten times or the freestyle at least three times without errors. Likewise, various questions were asked for each intervention, e.g., regarding fear, competence, etc., in terms of trampolining and ergometer riding, based on recordings using visual analogue scales ranging from 0 to 10.

Bicycle ergometer: The bicycle ergometer task served as a control task which results in a similar physical strain as the trampolining session, while consisting of a motor task already known to the participants. Hence, no motor learning should be involved during the control task. The sessions were of the same duration as on the trampoline in 10 min blocks. The bicycle session was performed at a moderate level of activity with a heartrate of 140 bpm at had an overall duration of 2 h.

Physical exertion was measured using Polar watches. Due to the balanced study design, it was not possible to make the load level on the ergometer (default in pulse rate) equal to the trampoline unit. In addition to the objective measurement of pulse rate, after each session the participants had to rate their perceived exertion using the Borg scale (Borg, 1973). This scale ranges from 6 = “very, very light” to 19 = “very, very heavy” and 20 = “exhaustion”.

### 2.4. Measurement

#### 2.4.1. Subjective Measurements of Sleep

Landeck Inventory for the assessment of sleep disorders (German version entitled: Landecker Inventar zur Erfassung von Schlafstörungen, LISST; Schürmann et al., 2001): The LISST includes questions regarding sleep-related breathing disorders, insomnia, narcolepsy, restless-leg-syndrome, and disturbances in the circadian rhythm. Furthermore, questions regarding subjective sleep quality and subjective performance capability are included.

Sleep questionnaire-A and sleep questionnaire-B (German version entitled: Schlaffragebogen-A and Schlaffragebogen-B, SF-A and SF-B; Görtelmeyer, 1986): The SF-A and SF-B are two independent measures for the description of the quantitative and qualitative assessment of sleep behaviour and the sleep experience. The SF-A refers to the past night and the SF-B to the past two weeks, respectively. 

#### 2.4.2. Objective Measurements of Sleep

Standard polysomnographic recording was performed according to Rechtschaffen and Kales (1968) during the whole night. Polysomnography included EEG (F3, F4, C3, C4, O2, O1), EOG, and submental EMG. EEG was recorded using an analogue polysomnogram (Model 4412P) from Nihon Koden (Irvine, CA, USA) or a polysomnogram from Schwarzer (ComLab32). Sleep stages were manually scored by an independent proficient rater according to Rechtschaffen and Kales (1968). Different sleep parameters were analysed in the study: time in bed (min), sleep efficiency (%), sleep latency (min), number of awakenings, awake % SPT, stage 1 % SPT, stage 2 % SPT and SWS % SPT, REM % SPT, REM latency (min), REM latency (3 min), duration of first REM period, and REM density (first REM period and whole night). REM density was rated manually ranging from 0 to 10 (no eye movement to high-density eye movement) [16].

### 2.5. Statistics

Statistical analysis included two-tailed *t*-tests for dependent samples, except for the REM sleep parameters where one-tailed *t*-tests for dependent samples were calculated. The statistical analyses were performed using IBM SPSS Statistics for Windows (version 15.0). The main question with respect to the original study [12] was on REM sleep; therefore, no correction for multiple comparisons was applied. 

Cohen’s d was used as the effect size and interpreted according to (Cohen, 1988) as small (≥0.2), medium (≥0.5), and large (≥0.8). A significance level of *p* < 0.05 was used for all of the inferential statistics.

## 3. Results

### 3.1. Subjective Sleep Parameters

Table 1 shows the subjective sleep parameters of the three nights of the experiment. There are no significant differences regarding the subjective sleep factors of the SF-A between the experimental and control night. 

### 3.2. Objective Sleep Parameters

None of the participants showed any medical peculiarities during the adaptation night. Table 2 shows the sleep parameters of the adaptation, control, and experimental nights. 

No significant differences were found in the NREM parameters between the control and experimental night. However, significant differences were found in the comparison of the REM sleep parameters of the control and experimental night. The REM % SPT was significantly higher during the experimental night than the control night, with a small effect (*t*(21) = −1.9, *p* = 0.04, d = 0.4). Furthermore, the REM density of the first REM period was higher during the control night (*t*(21) = 1.9, *p* = 0.04). No additional significant differences were found. 

### 3.3. Motor Learning and Control Task

Table 3 shows the results of various psychological variables after the control and the experimental condition, respectively. The participants felt significantly less boredom, more competence, less fear, and more control during the trampoline intervention.

Table 4 shows the physical variables during the intervention. The physical strain was higher during the trampolining session (*t*(19) = −4.7, *p* < 0.01), overall break duration was longer (*t*(19) = −5.6, *p* < 0.01), and a higher value on the Borg scale (*t*(20) = −2.3, *p* = 0.03) was reported during the trampolining session. 

Table 5 shows the descriptive performance of the various trampoline variables. 

## 4. Discussion

In the study at hand, we tested the influence of a trampolining motor learning session on the sleep architecture of the following night, in comparison with the sleep architecture following a control task on the bicycle ergometer. As hypothesised, we showed a significant increase in REM sleep after the motor learning task. No changes in sleep architecture were observed for NREM sleep parameters.

Observing a change in sleep architecture following a motor learning session and comparing it with a baseline night does make it possible to show the causal link between certain sleep stages and motor learning. However, until now only a few studies used this design with complex skill learning [5,6,7,8]. The results of these previous studies are hardly possible to compare with the study at hand. Previous studies either used a multitask strategy, which make it difficult to untangle the influence of one single motor task used [6], or otherwise used a daytime sleep design, which makes it hard to generalise the findings to a whole night of sleep, as daytime naps have an altered sleep architecture due to circadian and homeostatic effects [13]. The only study which is comparable is the study conducted by Erlacher and Schredl [5], in which the effects of sleep on sleep architecture were also investigated in a balanced within-design manner. However, our study contradicts Erlacher and Schredl’s findings, as no increase in REM sleep parameters was found. One obvious difference between the two studies is the used motor tasks with its different constraints [17]: While in both studies the focus was on difficulty maximation (in terms of accomplish different skill levels) in snakeboarding the goal is to move forward which requires rhythmical movements on the board and in trampolining one must orientate oneself in the three-dimensional space. Lately, the influence of the motor task and its various dimensions has been extensively discussed in the literature [3].

In the study at hand, we replicate previous finding by Buchegger (1993), while making some methodological changes to the original design. Firstly, in the Buchegger study, participants visited the laboratory three times during the 13-week training programme, which leads to a different level of expertise of the participants. This different level of expertise in turn can influence motor memory consolidation [18]. Furthermore, there are theories that link new learning as mainly dependent on REM sleep and that suggest Stage 2 sleep is important for optimisation [16]. Secondly, an adaptation night was introduced to consider the diminished REM sleep duration during the first night in the sleep laboratory [9]. Thirdly, a control task was performed during the control night which was supposedly free of the motor learning aspect but represented the physical load during the trampolining session. A combination of these three factors might have led to the diminished effect compared to the original study. However, even after controlling for these three factors, the effect reported by Buchegger (1993) still holds. Furthermore, different experimental approaches exist to investigate the effects of motor memory consolidation in sleep [3]. In addition to coordination and balance, the assessed trampolining task necessitates keen attention to visual information. The integration of these elements ensures a harmonious execution, emphasizing the multifaceted nature of trampolining. Successful performance relies on a dynamic interplay of physical and cognitive skills, highlighting the task’s complexity and demanding a holistic approach.

To compare the training session with a motor learning task, riding an ergometer was chosen as a control task as it is a physical activity with a physical load as similar as possible to that of trampolining and, at the same time, it is a familiar task, ensuring that no further motor learning processes occur. A moderate endurance training load was required, and it can be assumed that all of the test participants were able to ride a bicycle; thus, no motor learning processes would occur for any of the participants. The load times on the ergometer were staggered in 10 min blocks parallel to the trampoline units. The session on the bicycle ergometer was performed at a moderate power level—controlled by heart rate (140 Hz). However, in total, the physical load was higher during the trampolining compared to riding the ergometer. Even though the muscle recovery requirements might be higher for the trampoline task compared to the ergometer task, usually it is assumed that such sleep-related recovery processes are related with SWS rather than REM sleep [19]. The results showed no effects on SWS, and it could be concluded therefore that the additional physical load had no effect on the sleep parameters. However, in recent years, there have also been some therapies that have linked muscular recovery processes with REM sleep [20,21], so in future studies researchers should be even more careful that the physical effort in the motor learning tasks is the same.

The findings in this paper suggest that REM sleep increases following the acquisition of gross motor skills but to a lesser extent than found in previous studies. Longer REM sleep durations were associated with a decline in gross motor performance during subsequent nights’ sleep, resulting in reduced steering accuracy. REM sleep has been proposed as a neurophysiological marker of synaptic potentiation, reflecting plasticity-related changes in the cortico-striatal motor system after motor learning [16]. However, Saletin et al.’s (2011) [22] model highlights the dual role of sleep in strengthening and selectively forgetting human memories. Consequently, one could hypothesize that sleep may consolidate or erase new memories based on real-life relevance. Since learning trampoline seems not to interfere with the everyday skill, REM sleep might protect ecologically valuable memories. Similarly, it was found that sleep selectively enhances memory, focusing on the importance of learned material for future expectations [23]. Therefore, our data support the main conclusion that the brain consolidates information during sleep primarily for future relevance, which may not be the case for trampolining. Fischer et al. (2011) [24] investigated whether sleep benefits directed forgetting (suppressing retrieval of a dominant memory). They found that REM sleep, in particular, seems to counteract inhibitory control over dominant memories, making them more accessible for retrieval. Considering the dominant implicit (vs. explicit) learning component during trampolining, diurnal REM sleep might have similar effects in adapting the highly ingrained process of other motor tasks. Regarding the significance of diurnal sleep, Morita et al. (2012) observed improved performance in a complex motor skill learning task (three-ball cascade juggling) after sleep, compared to wakefulness. They noted an increase in EEG spectral power during N3 after juggling, which is known to be crucial for consolidating explicit knowledge. The authors suggested that even implicit tasks initially involve explicit memory systems, and complex motor skill learning, like juggling, may require more time to automatize processes, and thus involves a more extensive explicit process. Studies on memory consolidation for motor skills revealed that explicitly known new motor sequences require a functional interaction between the basal ganglia (striatum) and limbic (hippocampus) systems during post-training sleep [25]. Conversely, motor skills without explicit knowledge involve a distinct neural network, mainly the cerebellum and associated cortical regions, which appears to be less dependent on sleep [26]. In line with Robertson’s awareness theory [27,28], sleep may enhance learning gains only when subjects possess full explicit knowledge of the motor skill they are learning. Given that our innovative gross motor task is primarily implicit, the impact of sleep, especially during the early learning phase, may be relatively small.

Finally, it is interesting to note that we did not find an increase in REM density for the whole night; however, we did find a statistically significant increase in the first REM period. Since REM density is an index of REM sleep, we could have expected this to stay the same even if REM duration was increased. However, so far there is no clear evidence that an increase in the density or a certain pattern of eye movements indicates a role for eye movements in the learning of complex motor memories [16,29]. In a recent publication [30], it was shown that eye movements during phasic versus tonic rapid eye movement sleep might be seen as biomarkers of dissociable electroencephalogram processes for the consolidation of novel problems; therefore, in future studies, finer REM (and other microstructures of REM) but also NREM sleep analyses are needed [6].

The presented results also have important implications for sport practise. The presented results indicate that sleep is an important resource in the consolidation of motor memories. In particular, REM rich sleep in the morning is important and should not be interrupted. However, athletes seem to experience lower sleep quality than controls in periods of normal training [31] as well as before competitions [32]. 

## 5. Conclusions

The present study replicated the findings of Buchegger et al. [12] while adapting the experimental setup. Specifically, we optimized the learning protocol for one session, added an adaptation night, and introduced a physical control task with limited motor learning components. While our results do replicate Buchegger et al. [12], the statistical effects were smaller than in the original study. For sport practice, the presented results have important implications: REM sleep rich sleep in the morning plays an important role in motor memory consolidation and should therefore not be interrupted after sessions of complex motor skill learning.

## Figures and Tables

**Table 1:** Subjective sleep parameters for the adaptation, control, and experimental night (means ± SD).

Subjective Sleep Variables	No Task (Adaptation Night)	Bicycle Night (Control Night)	Trampoline Night (Experimental Night)	*t*-Test	
	M ± SD	M ± SD	M ± SD	*t*(18)	*p*
Sleep quality	2.83 ± 0.82	3.98 ± 0.40	3.88 ± 0.61	1.5	0.16
Feeling of being refreshed in the morning	2.95 ± 0.74	3.16 ± 0.69	3.15 ± 0.57	0.1	0.93
Balance in the evening	3.09 ± 0.76	3.60 ± 0.47	3.65 ± 0.44	−0.1	0.90
Fatigue in the evening	2.70 ± 0.75	3.42 ± 0.68	3.18 ± 0.63	1.3	0.21
Symptoms during sleep	1.64 ± 0.46	1.32 ± 0.31	1.33 ± 0.43	−0.2	0.88

**Table 2:** Global sleep architecture, NREM and REM sleep parameters for the adaptation, control, and experimental nights (means ± SD).

Objective Sleep Variables	No Task (Adaptation Night)	Bicycle Night (Control Night)	Trampoline Night (Experimental Night)	*t*-Test	
	M ± SD	M ± SD	M ± SD	*t*(18)	*p*
Global sleep architecture					
Total time in bed (min)	469.8 ± 23.3	471.0 ± 22.7	471.5 ± 11.0	−0.1	0.92
Sleep efficiency (%)	78.2 ± 21.9	89.7 ± 4.7	91.0 ± 3.9	−1.4	0.17
Sleep latency (min)	47.5 ± 89.4	18.2 ± 10.9	20.3 ± 12.8	−1.0	0.31
Number of awakenings	19.5 ± 10.2	18.3 ± 9.5	19.5 ± 8.7	−0.9	0.37
NREM parameters					
Wake % SPT	11.6 ± 15.6	6.2 ± 4.5	4.5 ± 2.5	1.9	0.07
Stage 1 % SPT	6.6 ± 3.6	8.1 ± 3.8	7.4 ± 2.4	1.1	0.29
Stage 2 % SPT	51.3 ± 13.2	53.7 ± 8.8	53.9 ± 8.9	−0.2	0.88
Stage 3 % SPT	18.7 ± 11.0	16.5 ± 9.2	17.5 ± 9.7	−1.1	0.27
REM parameters ^1^					
REM % SPT	11.7 ± 5.2	15.3 ± 4.7	16.5 ± 4.4	−1.9	0.04
REM latency (min)	136.2 ± 60.3	94.8 ± 38.2	97.4 ± 41.3	−0.3	0.38
REM latency (3 min)	150.6 ± 61.8	141.3 ± 58.7	139.3 ± 61.9	0.1	0.46
Duration of first REM period	13.3 ± 8.8	12.7 ± 11.9	12.9 ± 9.9	−0.1	0.47
REM density (1. REM period)	8.5 ± 6.3	9.7 ± 5.6	7.6 ± 4.6	1.9	0.04
REM density (whole night)	11.9 ± 4.2	12.1 ± 4.8	12.2 ± 4.9	−0.1	0.47

Note: ^1^ probability values are one-tailed.

**Table 3:** Subjective ratings for the control and experimental night (means ± SD).

	Bicycle Night (Control Night)	Trampoline Night (Experimental Night)	*t*-Test	
	M ± SD	M ± SD	*t*(21)	
Feeling comfortable	8.64 ± 3.00	10.25 ± 3.08	−1.8	0.08
Boredom	7.39 ± 4.28	1.39 ± 2.85	6.0	<0.01
Variety	3.36 ± 2.96	10.73 ± 2.76	−8.9	<0.01
Competence	10.75 ± 2.15	8.00 ± 2.69	4.2	<0.01
Fear	0.39 ± 0.58	2.73 ± 2.75	−3.9	<0.01
Control	6.00 ± 4.45	11.48 ± 1.70	−6.3	<0.01

**Table 4:** Subjective and objective physical strain during the intervention (means ± SD).

	Bicycle Night (Control Night)	Trampoline Night (Experimental Night)	*t*-Test	
	M ± SD	M ± SD	*t*(20)	
Physical strain	141.70 ± 6.24	157.66 ± 14.54	−4.7	<0.01
Break	97.53 ± 10.36	115.14 ± 14.17	−5.6	<0.01
Borg	13.55 ± 1.84	14.95 ± 1.96	−2.3	0.03

**Table 5:** Descriptive data on learning success on the trampoline (means ± SD).

Variables	Results
Number of elements	7.59 ± 1.44
Time to turn jump	39.34 ± 7.90
Time to first long programme	46.93 ± 8.57
Time to second long programme	51.60 ± 6.50

## Data Availability

All relevant data are within the paper.
